# A Pilot Feasibility Trial of Multi-Program Epidural Spinal Cord Stimulation for Bladder Function Recovery in Chronic Spinal Cord Injury

**DOI:** 10.3390/jcm15166287

**Published:** 2026-08-14

**Authors:** Charles H. Hubscher, Siqi Wang, Terri Manning, Claudia A. Angeli, Maxwell Boakye, Pawan Sharma, Jordan Matelsky, Breanne Christie, Erik Johnson, Francesco Tenore, Susan J. Harkema

**Affiliations:** 1 Kentucky Spinal Cord Injury Research Center, University of Louisville, Louisville, KY 40202, USA; siqi.wang@louisville.edu (S.W.); max.boakye@louisville.edu (M.B.); 2 Department Anatomical Sciences and Neurobiology, University of Louisville, Louisville, KY 40202, USA; 3 Tim and Caroline Reynolds Center for Spinal Stimulation, Kessler Foundation, West Orange, NJ 07052, USA; cangeli@kesslerfoundation.org (C.A.A.); psharma@gsrh.org (P.S.); sharkema@kesslerfoundation.org (S.J.H.); 4 Department of Physical Medicine & Rehabilitation, Rutgers New Jersey Medical School, Newark, NJ 07103, USA; 5 Department Neurological Surgery, University of Louisville, Louisville, KY 40202, USA; 6 Research and Exploratory Development Department, Johns Hopkins Applied Physics Laboratory, Laurel, MD 20723, USAfrancesco.tenore@jhuapl.edu (F.T.)

**Keywords:** spinal cord injury, neuromodulation, epidural stimulation, bladder, external urethral sphincter

## Abstract

**Background/Objectives:** Spinal cord epidural stimulation (scES) holds significant potential for restoring autonomic function after chronic spinal cord injury (SCI). A significant and often debilitating sequela of SCI is neurogenic lower urinary tract dysfunction, characterized by loss of voluntary bladder control, which adversely impacts patients’ dignity, urological health, and overall quality of life. In a pilot study of three individuals with SCI, an epidural electrode array was implanted between L2 and sacral segments to modulate lower urinary tract function for efficient emptying. **Methods**: Three participants with chronic thoracic clinically complete SCI were implanted with an epidural stimulator over the lumbosacral spinal cord. Array placement was confirmed using intraoperative and postoperative spatiotemporal mapping of known motor neuron pools following published protocols. In a controlled laboratory mapping, bladder storage and voiding neural networks were targeted during filling cystometry by interactively optimizing lower urinary tract stimulation parameters (electrode configuration, frequency, intensity, and pulse width). Efficacy was assessed during six-hour laboratory sessions of natural bladder filling, followed by approximately four months of daily at-home use. **Results:** Pre-implant cystometry revealed poor voiding efficiency and absent bladder sensation. Following bladder scES mapping and home training, one of the three participants regained direct bladder sensation, exceeded the 90% clinical threshold for efficient voiding, and continues to use scES for bladder management instead of intermittent catheterization, with no urinary tract infections reported since completion of training (February 2022). The inability of two participants to attain high voiding efficiency may be due to suboptimal adherence to prescribed bladder training protocols in one case and the presence of an elevated bladder neck in the other. All three participants reported off-target improvements in bowel and sexual functions. **Conclusions**: The present novel pilot study provides the first evidence supporting the potential use of epidural stimulation for bladder management and the importance of activity-based recovery training for durable performance.

## 1. Introduction

Current therapies to improve daily bladder management (storage/emptying) after spinal cord injury (SCI) include catheterization, pharmacologic and surgical interventions, and functional electrical stimulation [1,2]. Techniques that target function, such as electrical stimulation of spinal nerve roots, peripheral nerves or the peripheral organ itself [3,4,5,6,7,8], have been applied but have not led to widespread clinical use due to limited effectiveness or the irreversible invasive nature of the approach. Spinal cord epidural stimulation (scES) post-SCI has shown great promise for restoration of voluntary and spinally mediated bladder control [9,10,11,12,13].

Clinical interventions that promote efficient emptying with low residual volumes remain elusive. Having post-void bladder residual volumes is important to steer clear of repeated urinary tract infections, to avoid urinary bladder stones due to accumulation of sediment that crystallizes and accumulates, and to prevent chronic vesicoureteral reflux and hydronephrosis due to high intravesical pressures [14,15]. Previously conducted clinical and pre-clinical (rat) studies used targeted scES to improve bladder capacity and void initiation with minimal concomitant motor responses [9,16,17,18,19,20].

Although current scES devices were initially designed and approved to treat neuropathic pain [21] and have been implanted in thousands of individuals with a very low rate of procedural complications [22], technological limitations exist for use in SCI. For the current pilot trial, recovery of bladder function was examined in a few individuals with chronic clinically motor- and sensory-complete paralysis using neuromodulation of bladder pathways in the home environment with more recent technology that transitions scES for storage (bladder capacity—BC-scES) to emptying (bladder void—BV-scES) in accordance with real-time physiological responses (such as sensations of fullness or urgency).

## 2. Methods

### 2.1. Participant Recruitment and Screening

The recruitment process was performed through our secure research database, which includes over 6000 individuals registered with SCI. Potentially eligible research participants were invited for an information session to discuss the complete protocol, including risks and benefits, with the principal investigator and/or designated research staff. The recruitment goal for the pilot trial was four participants. The study was registered on ClinicalTrials.gov (NCT03452007). Data and experimental protocols associated with this study are available on the SPARC Portal (RRID: SCR_017041): https://doi.org/10.26275/t2ve-vu9o [23].

Each potential research participant was screened for medical eligibility by a study physician and for scientific eligibility by the principal investigators. Inclusion criteria were male and female adults; stable medical condition; American Spinal Injury Association Impairment Scale (AIS) classification A or B; presence of neurogenic bladder dysfunction; clear indications that the period of spinal shock is concluded as determined by the presence of muscle tone, deep tendon reflexes or muscle spasms, and discharge from standard inpatient rehabilitation; at least two years post-SCI; and non-progressive supra-sacral SCI. Exclusion criteria were botulinum toxin injections of the bladder within the past year and/or bladder augmentation surgery.

After study eligibility was determined and consent procedures implemented, the study physician completed a medical history and neurological examination for each SCI individual for medical eligibility. All research participants signed an informed consent that had been approved by the University Institutional Review Board (IRB) prior to entering the study. After eligibility was determined and consent procedures implemented, each participant underwent screening assessments (urodynamics/cystometry with blood pressure monitoring and bladder/kidney ultrasound) to determine baseline lower urinary tract morphology and function. The International SCI Data Set Questionnaires for bladder [24], bowel [25] and sexual [26] functions were completed at screening, then again post-training and at follow-up.

### 2.2. Positioning and Testing scES Array During Implantation

**Figure 1:**
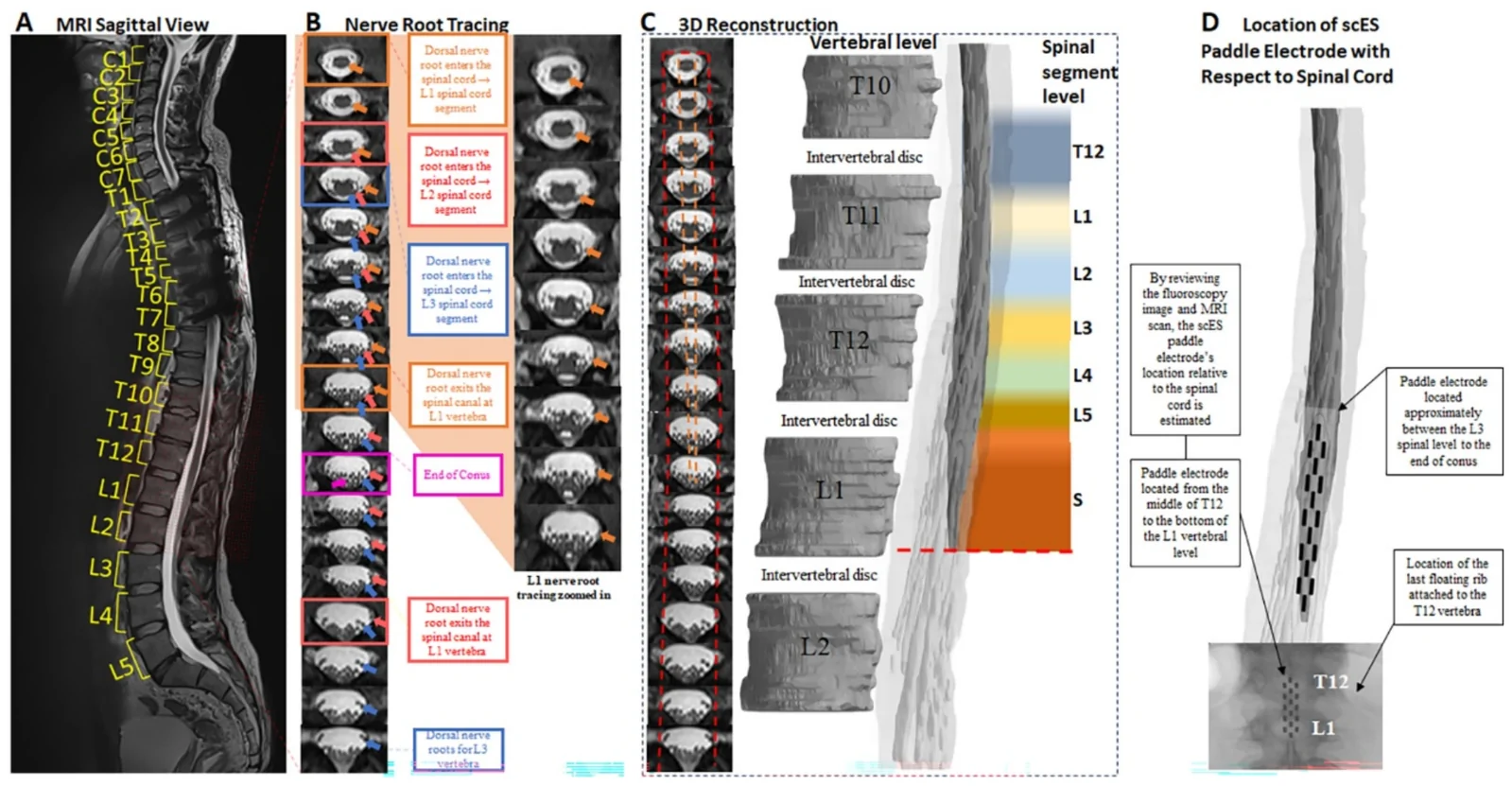
Example of neuroanatomical mapping of the spinal cord at lumbosacral enlargement (Participant ID-A70). (**A**). MRI sagittal view of the whole spine in an individual with a thoracic spinal cord injury, with the individual vertebral body indicated in yellow. (**B**). Axial images of the lumbosacral region from a high spatial resolution MRI recording, with the arrows pointing at the dorsal nerve roots from the vertebral site of the exit back to the location of entering the spinal cord to show how spinal cord levels are identified using the nerve root tracing technique. (**C**). Process of tracing and labeling the spinal cord region (orange dashed lines), cerebrospinal canal (red dashed lines) and nerve roots in each axial image to build a reconstructed model of the lumbosacral spinal cord from the segmented images along with associated vertebral bodies and spinal segment levels. (**D**). Process of integrating intraoperative fluoroscopy images of the paddle placement with the preoperative MRI recordings to estimate the location of the paddle array on the spinal cord model.

Following previously published protocols [27,28,29,30], participants had a 16-electrode array lead implanted epidurally over lumbosacral spinal segments and a spinal stimulator implanted on the right or left flank. An example from one participant is provided in Figure 1. The electrode paddle was inserted over the midline of the exposed dura and positioned based on MRI-based spinal cord modeling [31] (Figure 1) and the activation threshold and timing of leg muscles using 2 Hz stimulation, as detected by electromyography (EMG) recording electrodes (Supplementary Figure S1A). EMG data were recorded with surface electrodes placed over the muscles, apart from wire electrodes in the iliopsoas muscles. After positioning the epidural electrode array, wire leads were tunneled subcutaneously to the flank and connected to the stimulator. Multiple bipolar stimulation configurations were tested with use of midline and left–right electrode pairs within the array (Supplementary Figure S1B).

### 2.3. Spatiotemporal and Cardiovascular Mapping

**Figure 2:**
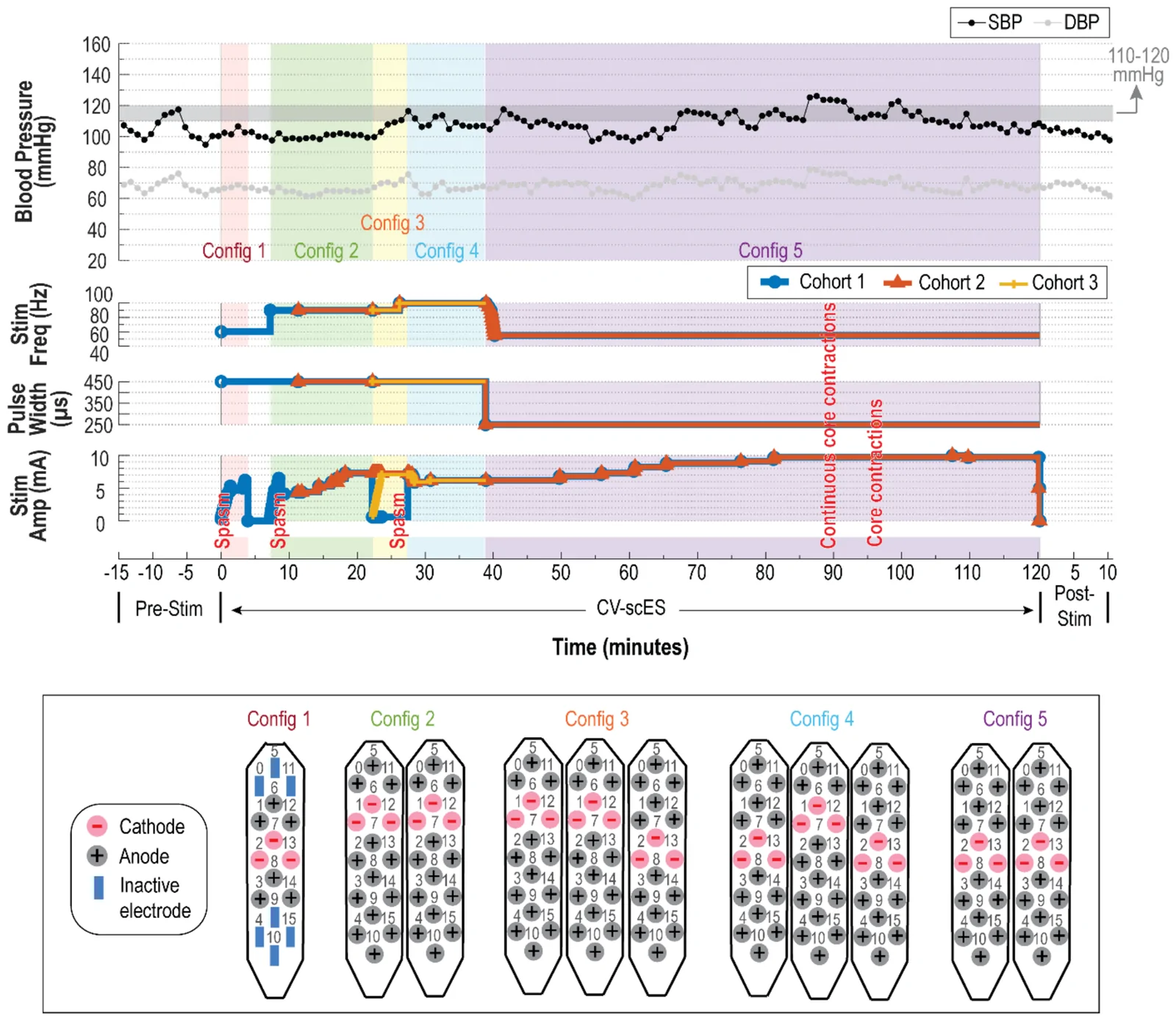
Example of CV-scES mapping for blood pressure stabilization (Participant ID-A70). Systolic blood pressure (black circles, mean of 1 min beat-to-beat series) and diastolic blood pressure (gray circles) during 15 min Stim OFF before mapping (pre-stim), 2 h CV-scES mapping (CV-scES) and 15 min Stim OFF after mapping (post-Stim) are shown. The gray horizontal strip indicates the target range of systolic blood pressure of the CV-scES mapping, 110–120 mmHg. The shaded colors represent the time periods of stimulation with different configurations (Config): Config 1 in red, Config 2 in green, Config 3 in yellow, Config 4 in blue and Config 5 in purple. The stimulation frequency (Stim Freq), pulse width, and stimulation amplitudes (Stim Amp) are shown below the blood pressure (Cohort 1: blue circles and line; Cohort 2: orange triangles and line; Cohort 3: yellow bars and line). The time axis denotes the time when spasms and core contractions occurred, and blood pressure was affected. The anodes and cathodes, stimulation frequency and pulse width of each configuration are shown at the bottom. From Config 1 to Config 5, the cathode locations were tested along the rostral–caudal direction, the number of anodes was increased, and the number of cohorts was increased or decreased to optimize the sensitivity of the blood pressure responses and/or avoid muscle activity. CV-scES: spinal cord electrical stimulation optimized for cardiovascular control, i.e., blood pressure stability.

After about a three-week postsurgical period for healing around the electrode array and flank incision, EMG was used in an initial session to identify electrode location relative to known motor neuron pools while the research participant was supine [28,32] (see Supplementary Figures S2 and S3). In a subsequent session, blood pressure was measured continuously using plethysmography (Finometer Pro and Finapres NOVA, FMS, Enschede, The Netherlands) and an automated sphygmomanometer (Welch Allyn, Skaneateles Falls, NY, USA) while evaluating the impact of various electrode combinations and stimulation parameters (example provided in Figure 2) that provided the most stable blood pressure (systolic 110–120 mmHg).

### 2.4. Bladder Management with Epidural Stimulation

Specialized research software developed for modulating motor and autonomic function (Medtronic STIMX 2.0) enabled use of multiple configurations during filling cystometry. STIMX 2.0 enabled four configurations (cathode, anode selection; frequency; pulse width; and amplitude) in each program (bladder storage, bladder emptying) that could be sequenced within milliseconds, directly transitioning the electrical fields. Each configuration within the program was deemed a “cohort” (per software terminology) that would focus on bladder capacity (BC-scES) or voiding (BV-scES), and cardiovascular control (CV-scES). In addition to the cardiovascular cohort, spinal interneurons linking pelvic inputs and hypogastric outputs [33] were targeted for bladder storage (BC-scES, detrusor relaxation and internal urethral sphincter closure). For efficient emptying (BV-scES), the sacral spinal micturition neural network was targeted for void initiation (pelvic nerve reflex circuitry for bladder contraction) in combination with the purported L3 lumbar spinal coordinating center for neuromodulation of the external urethral sphincter (EUS) [33,34,35].

Evaluation of scES for daily bladder management was first assessed in a controlled laboratory setting during filling cystometry by interactively identifying maximally effective stimulation cohorts for both storage and emptying [16,32] while integrating the cardiovascular cohort. For all participants, urethral and rectal catheters used were both size 7Fr, and the infusion rate was 20 mL/min. The detrusor and urethral pressure responses and EUS EMG responses were monitored during both storage and emptying phases (void attempts) as previously described [16] to identify cohorts for bladder compliance and voiding efficiency ([Void Amount/(Void + Residual)] × 100) at safe pressures per published guidelines of the International Continence Society [36]. If needed, a cohort for spasticity was also integrated into the bladder program. Blood pressure and heart rate were recorded during mappings using plethysmography (Finometer Pro and Finapres NOVA, FMS, Enschede, The Netherlands; Human NIBP Nano Interface by ADInstruments, Colorado Springs, CO, USA) and an automated sphygmomanometer. The participant was instructed to communicate bladder sensations (i.e., sensation, desire, urgency) and any symptoms of autonomic dysreflexia. The efficacy of the programs for capacity and voiding was then assessed for six hours in the laboratory setting during two fill–void cycles with natural bladder filling. Once the participant demonstrated proficiency and skill in software operation, the programs for capacity and voiding were made available on a dedicated tablet for at-home application for approximately 4 months for use of up to 8 h a day (2 to 3 fill–void cycles depending on intake) for at least 5 days per week. The participant performed daily call-ins and was brought back for urodynamic testing after approximately 20 at-home sessions, after the last session, and at six- and twelve-months post-study for follow-up.

Using a within-subjects study design, the primary feasibility pilot study outcome was pre-specified as percent change in void efficiency, which accounts for post-void residual volume. To provide evidence supporting the effectiveness and feasibility of the scES intervention, post-mapping pre-training data were compared within subjects against baseline, post-training and follow-up time points. The Data Set Questionnaires were secondary outcomes. An additional secondary outcome was effects of bladder filling and emptying on systolic blood pressure without versus with CV-scES. Comparisons of void efficiency among pre-training with scES OFF, pre-training with scES ON and post-training with scES ON were made with recordings of home-training sessions (Participants A70 and A124 only). A mixed-effects model was used to test the main effects of participant and time point/stimulation condition, as well as the interaction between participant and time point/stimulation, with fill–void cycle as a random effect. Post hoc analyses were performed with Tukey’s multiple comparisons test, comparing the effect of time point/stimulation condition on void efficiency in each participant.

## 3. Results

### 3.1. Participant Characteristics

Four individuals with SCI consented to the study. Two participants completed the study through the twelve-month follow-up. One individual withdrew from the study just prior to the six-month follow-up assessments, while another withdrew prior to implantation due to external issues related to relocation and work. The demographics for all four individuals are provided in Table 1. The AIS was used to classify completeness of SCI [37]. The spinal cord segmental level of injury was identified by the highest level above which there was normal motor and sensory function. The scores obtained prior to the array implantation are also provided in Table 1. All participants used intermittent catheterization for their daily bladder management. None of the participants presented with bladder or renal disease unrelated to SCI, and none received prior bladder botulinum toxin injections. Renal bladder ultrasound before surgical implantation (N = 3) showed no evidence of hydronephrosis, nephrolithiasis, or mass lesions and indicated a grossly normal bladder with no evidence of intraluminal masses or wall thickening (Table 2). The abdominal aorta was also grossly normal. Filling cystometry pre-implant indicated the presence of low voiding efficiency and absence of bladder-related sensations.

### 3.2. Bladder scES Mapping

The cathode selection was determined by the electrode position relative to spinal level based upon the 3D spinal cord reconstruction (see Figure 1 example). Obtaining a cardiovascular cohort (CV-scES) in advance of bladder mapping (see Figure 2 example) was a necessary step for safety given that bladder fullness post-SCI is a common trigger of autonomic dysreflexia (a sudden sharp rise in blood pressure with intolerable symptoms) [38,39,40] and is a limiting factor to attain storage of urine within the normal physiological range (300–600 mL).

**Figure 3:**
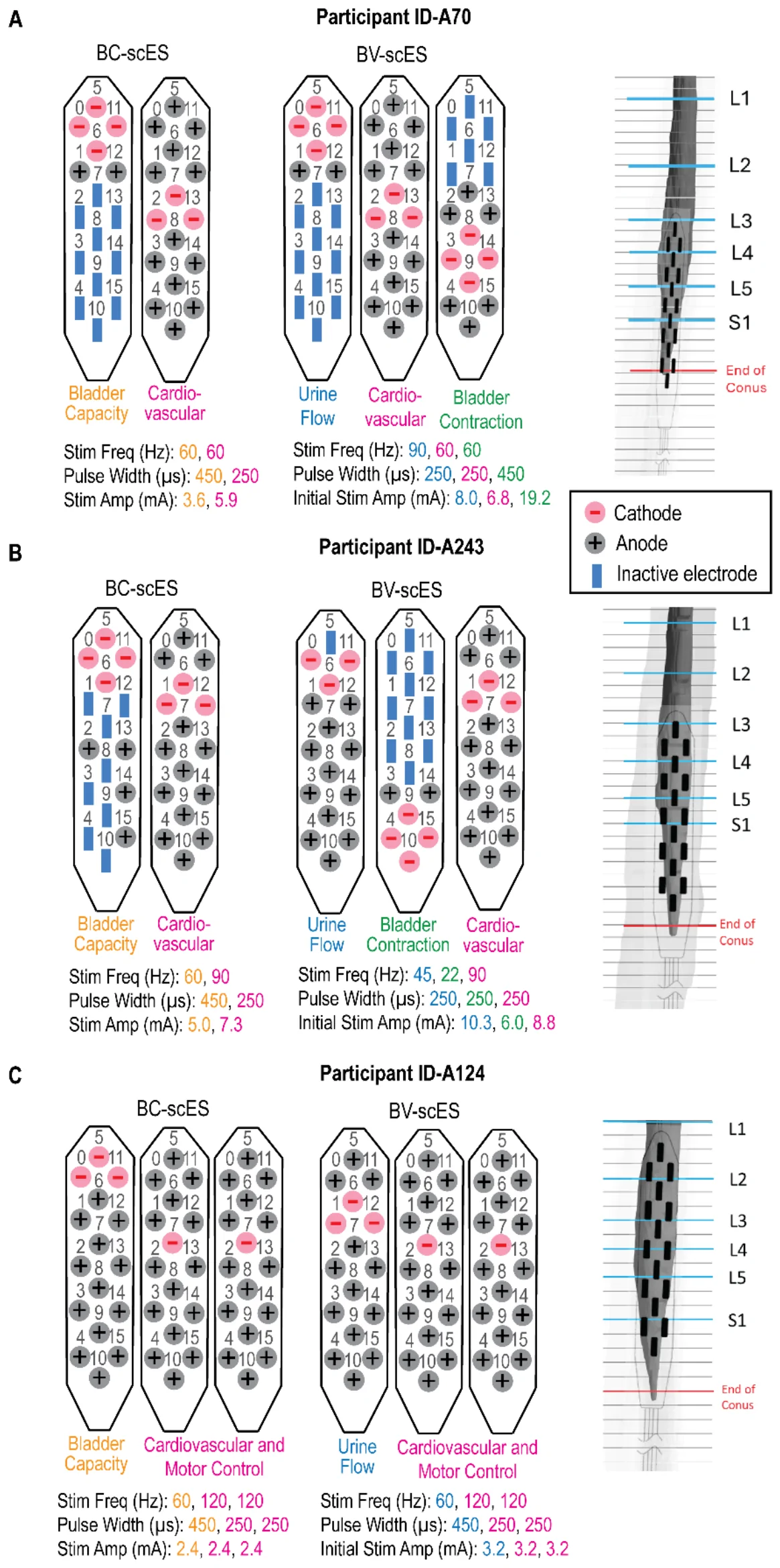
Neuromodulation Mapping Summary. Configurations of spinal cord epidural stimulation (scES) and the locations of the implanted electrode paddles in Participants ID-A70 (**A**), ID-A243 (**B**) and ID-A124 (**C**). For each participant, there were two sets of interleaving cohorts of cathode–anode combinations, i.e., programs: (1) the program targeting bladder capacity (BC-scES), shown on the left, and (2) the program targeting voiding (BV-scES), shown in the middle. The targeted functions of the interleaving cohorts of cathode–anode combination are labeled under each cohort, with stimulation frequency (Stim Freq), pulse width and initial stimulation amplitude (Stim Amp) displayed beneath the sets. The amplitudes of BC-scES did not change during the capacity phase in all participants, and the amplitudes of BV-scES increased to 9.2, 6.8 and 20 mA in Participant ID-A70, to 17.8, 11 and 8.8 mA in Participant ID-A243, and to 6.2, 6.2, and 6.2 mA in Participant ID-A124. Red circles with a minus sign indicate cathodes; black circles with a plus sign indicate anodes; blue rectangles indicate inactive electrodes that did not inject any electrical current. Color-coded cohorts for different functional targets are illustrated in the programs order of stimulation, as the sequence impacts the electrical field properties. The overlay of the electrode paddle and 3D reconstruction of spinal cord MRI is shown at the right. Horizontal lines correspond to axial MRI images. The spinal levels are indicated beside the image with blue lines indicating lumbar (L) to sacral (S) levels and red line indicating the end of conus.

**Figure 4:**
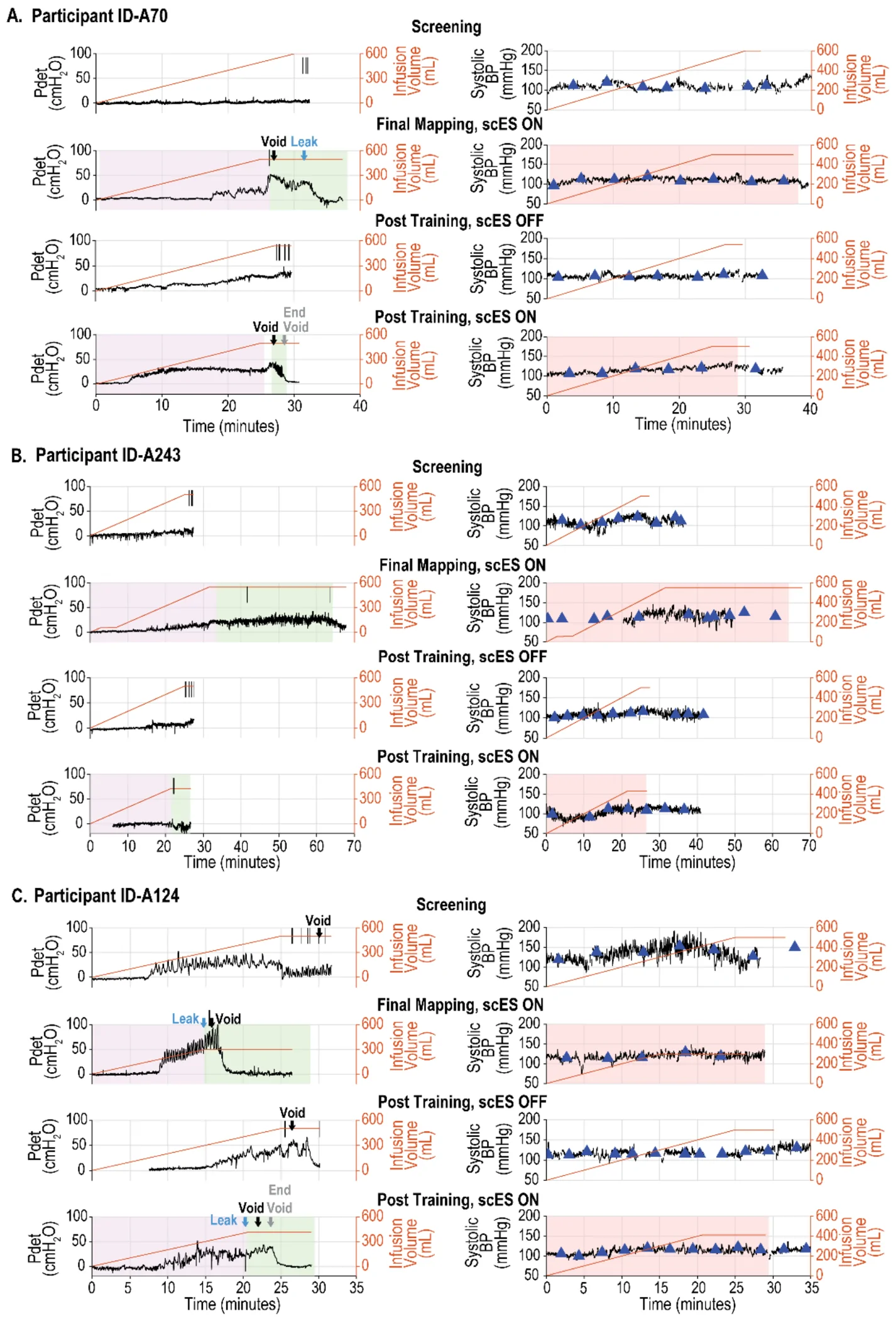
Longitudinal assessment. Detrusor pressure (Pdet) and systolic blood pressure (BP) during Urodynamics of Research Participants ID-A70 (**A**), ID-A243 (**B**) and ID-A124 (**C**). Pdet is shown in the left column in black; infusion volumes are shown in orange. Purple areas indicate activation of scES for bladder capacity and green areas indicate activation of scES for voiding. Beat-to-beat systolic BP is shown in the right column as black lines; brachial systolic BP is shown as blue triangles; infusion volumes are shown in orange. Red areas indicate activation of scES for blood pressure stability. In (**A**), four assessments are shown at pre- and post- training time points, without stimulation (No Stim) or with spinal cord epidural stimulation (scES): screening with No Stim (94 days before implantation), final mapping with scES (75 days after implantation), post training with No Stim (220 days after implantation), and post training with scES (222 days after implantation). In (**B**), four assessments are shown at pre- and post-training time points with No Stim or with scES: screening with No Stim (127 days before implantation), final mapping with scES (159 days after implantation), post-training with No Stim (404 days after implantation), and post-training with scES (385 days after implantation). In (**C**), four assessments are shown at pre- and post-training time points, with No Stim or with scES: screening with No Stim (15 days before implantation), final mapping with scES (224 days after implantation), post-training with No Stim (439 days after implantation), and post-training with scES (474 days after implantation). In all panels, black arrows indicate the start of voiding; gray arrows indicate the end of voiding if identified; blue arrows indicate leaking; black vertical lines indicate the start of void attempts; gray vertical lines indicate the end of void attempts if identified.

The detrusor and urethral pressure responses and EUS EMG responses were monitored during both storage and emptying phases (void attempts) during in-lab filling cystometry to identify optimal scES cohorts that would shift detrusor compliance and void efficiency ([Void Amount/(Void + Residual)] × 100) at safe pressures toward the normal physiological range per International Continence Society criteria [36] (efficiency greater than 90%). The configurations identified after 11–12 bladder mapping sessions for each of the three participants are presented in Figure 3. Note that each of the programs for storage and emptying of the bladder contains two or three cohorts, targeting different functions of the spinal circuitry. After in-lab evaluation during natural filling and confirmation of the participant’s proficiency in the operation of the software on a dedicated tablet, daily at-home training was initiated. A summary of the bladder function cystometry data from the different study time points (screening, post-mapping, post-training) and the recordings is provided for all participants in Table 3 and Figure 4, respectively. A summary of lower urinary tract data obtained through the International Data Set Questionnaire at screening, post-training, and follow-up time points are presented in Table 4.

### 3.3. Bladder scES Training Outcomes

#### 3.3.1. A70

**Figure 5:**
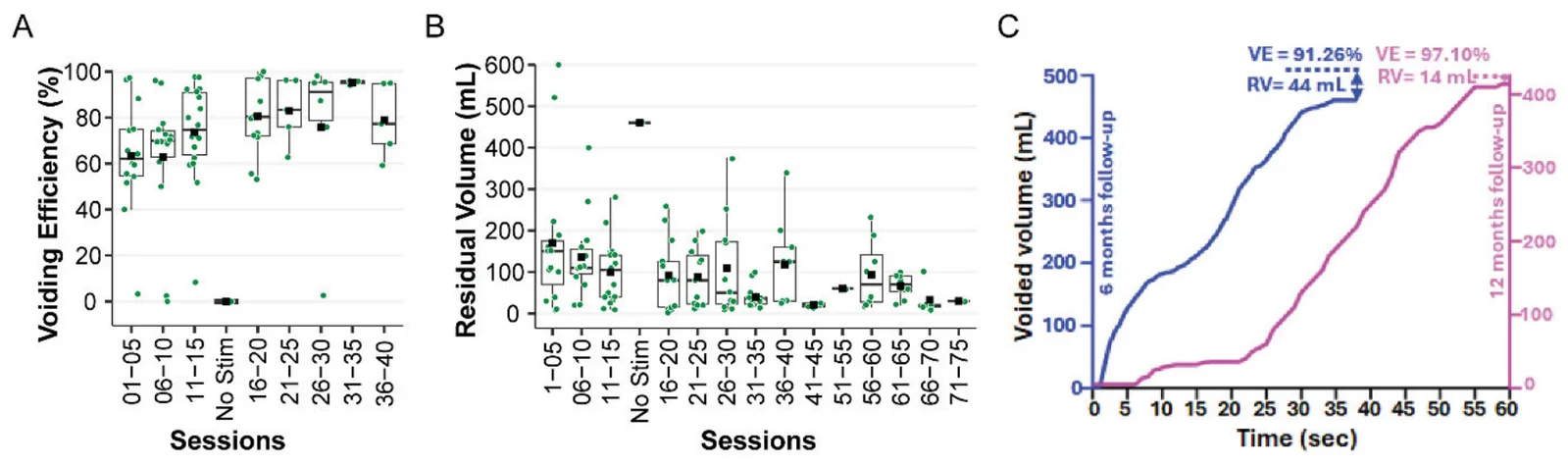
Improvement in ability to void with targeted scES (Participant A70). (**A**,**B**). At-home data showing increasing void efficiency (**A**) and decreasing residual volume (**B**) over the course of 75 daily sessions (every 5 sessions were grouped and plotted). Green circles indicate attempts to void using scES. The horizontal lines on the boxplot indicate the median; black squares indicate the mean; the boxes extend to the first and the third quartile; the whiskers extend to the maximum and minimum values within 1.5 * inter-quartile range. A void attempt with no scES (No Stim) on the day of Session #12 was included after Session 11–15. (**C**). A uroflow (void attempt without a catheter in place) was performed at the 6- and 12-month follow-up. Void efficiency was 91.26% at 6-month and 97.1% at 12-month follow-up, within the International Continence Society target of 90% or greater. RV—residual volume; VE—voiding efficiency.

Using in-lab filling cystometry (500 mL), voiding efficiency was substantially improved after bladder scES training, reaching 76%, compared with 15% pre-training with scES using the same cohort and 0% without bladder scES (Figure 4A). Participant A70 consistently had high voiding efficiency when using stimulation at home (75 days of at-home use for 1–3 fill–void cycles per day; Table 5), measuring low residual volumes post-void (Figure 5A,B; Table 5), without experiencing autonomic dysreflexia. Void efficiency significantly changed pre-training with scES ON from scES OFF, with an increase of 58.1% void efficiency (95% confidence interval (CI) [37%, 79%], *p* < 0.0001). The void efficiency at home was 0% for all fill–void cycles with scES OFF. Void efficiency of A70 changed further after training (Table 5) with an additional increase of 26.5%, from pre- to post-training with scES ON (95% CI [7%, 46%], *p* = 0.0100). The effects were sustained after continuous use at six- and twelve-month follow-up (Figure 5C), with the participant reporting he continues to use bladder scES solely for daily management. Voiding efficiency was higher during laboratory uroflow testing at follow-ups (91% and 97%) than during cystometry, which are not equivalent conditions due to the presence of a catheter and less favorable body positioning for cystometry. His at-home bladder management routine includes BC-scES for fill phase while performing activities of daily living and transferring to the toilet once sensory cues indicate bladder fullness (not present prior to study). Once on the toilet, he switches programs from BC-scES to BV-scES for emptying (as documented in in-lab uroflow testing in Figure 5C). Noteworthy is that no urinary tract infections were reported between the end of study and the twelve-month follow-up. In addition, ultrasound around the six- and twelve-month follow-up to assess the kidneys, bladder and abdominal aorta indicated no changes relative to pre-implant (Table 2). Note that the bladder scES program parameters were not adjusted during the training phase and follow-up.

#### 3.3.2. A243

Sensations of fullness associated with the need to void were reported after training with bladder scES. Sensations of urgency occurred while using the BV-scES program, which were not present either without stimulation or when the BC-scES program was in use. However, at no point during mapping was there a void with any attempts (done in sitting position), not even a small leak (Figure 4B). The participant reported that leaks would only occur during his bowel program while performing digital stimulation. A more in-depth clinical evaluation (cystourethroscopy) subsequently revealed an elevated bladder neck suggestive of primary bladder neck obstruction, a form of bladder outlet obstruction [41]. Participant A243 noted that after failing to void with BV-scES during home training, the rate of urine flow was substantially faster while catheterizing (relative to when not using stimulation). Note that ultrasound around the six- and twelve-month follow-up to assess the kidneys, bladder and abdominal aorta indicated no changes relative to pre-implant (Table 2). Note that the bladder scES program parameters were not adjusted during follow-up.

#### 3.3.3. A124

Using in-lab filling cystometry (500 mL), voiding efficiency was substantially improved after bladder scES mapping (pre-training), reaching 62.6%, compared with 1.8% at baseline without stimulation (Figure 4C). Note that due to a high level of muscle activity (spasms) during bladder filling and emptying, electrode selection and parameters were modified within the cardiovascular cohort to additionally target muscle relaxation (designated as Cardiovascular and Muscle Control in Figure 3C). Participant A124 had inconsistent voiding amounts when using BV-scES at home (Table 5). Three-day gaps (range of 1–10 days) occurred on average amongst consecutive training days (mean of 1.5 consecutive days of at-home use, with two fill–void cycles per day), indicative of non-compliance with instructions provided. A calculation of a training compliance factor (sessions [#] ÷ duration [days]) yielded a substantially low numerical value (0.242), suggestive of poor compliance with the study protocol. In contrast, Participant A70 had a compliance factor of 0.638. Void efficiency improved with scES alone (Table 3) but reverted backward from pre- to post-training with scES ON, with a decrease of 13.5% (95% CI [4%, 23%], *p* = 0.0083), without consistent training (Table 5). Note that for A124, statistical analysis did not include home data with scES OFF due to an absence of recording at home with scES OFF.

### 3.4. Off-Target Effects (Exploratory)

Prior to scES implantation, the International SCI Data Set Questionnaires revealed Neurogenic Bowel Dysfunction (NBD) scores of mild (A70), severe (A243) and moderate (A124) (Table 6) and the presence of sexual dysfunction (orgasmic function score re ejaculation ability—score of 2 is almost never or never) (Table 6). Noteworthy is that while the pilot study was being conducted, participants were not directed to make any changes to their daily routine, activities, and management for bladder, bowel, and sexual function (except while recovering from implant surgery). Thus, for example, the sexual function scores pertaining to erectile function prior to implant and thereafter for two participants are within the normal range due to medication use as part of their standard of care.

Bowel and sexual function questionnaires indicated desirable off-target improvements after home training with bladder scES and follow-up (Table 6). For bowel function, all three participants reported reductions either in terms of duration or amount of digital stimulation necessary for effective evacuation of stool. All participants also reported changes in erectile function, and two had improvements with ejaculation. Participant A70, unable to ejaculate since injury even with penile vibratory stimulation (attempted years earlier at a specialized clinic), reported being able to ejaculate in approximately 50% of attempts. All three participants report a lack of erections with scES ON. Participant A243 reported turning scES ON to counter TriMix-induced erection.

### 3.5. Adverse Events

Nine adverse events occurred over the course of study enrollment that all resolved within several days or weeks. Three for Participant A70 were not study-related (COVID; abnormal liver function test (LFT) levels; feeling cold unrelated to fever) and two others for Participant A70 were possibly study-related (increased urine volume and nocturnal leaking shortly after implant prior to mapping phase). For Participant A243, one possibly related adverse event occurred (participant-reported back popping with spinal rotation). A spinal x-ray indicated no changes to the stimulator position, and the neurosurgeon cleared the participant to resume research activities (occurrence during the study mapping phase).

There were three adverse events for Participant A124. Stimulation was paused during one mapping session as Participant A124 reported that spasms being experienced made breathing difficult (deemed possibly related to the study), which triggered feelings of anxiety that the participant related to a prior experience. The anxious feelings had a ripple effect on the next two mapping sessions before resolving. Vitals remained within normal limits, and the remaining mappings continued uninterrupted. A second adverse event that was categorized as definitely related to the study occurred at home during the mapping study phase when Participant A124 unintentionally turned on the stimulator when charging his device. It was noticed promptly, as a substantial leak was triggered, as the last configuration tested was for void. The stimulator was immediately turned off, which stopped the flow of urine. Future safeguards put in place included additional education on the handling of the device as well as removal of all cohorts during the mapping study phase so that even if a button was pressed, no stimulation would come on. A third adverse event, categorized as possibly related to the study (per study Data Safety Monitoring Board recommendation due to unknown cause), occurred at the post-training time point. The renal ultrasound revealed Participant A124 had a renal cyst. The participant was referred to his personal urologist for follow-up.

## 4. Discussion

All three pilot study participants incurred substantial benefits with use of bladder scES, which included the ability to sense bladder fullness or urgency as well as off-target effects on bowel and sexual function. Participant A70 was also able to consistently achieve almost complete emptying of the bladder with bladder scES training, permitting him to transition from daily intermittent catheterization to bladder scES-assisted voiding. These unprecedented breakthroughs were facilitated by the versatility of the implanted multi-electrode array, which enabled activation of multiple spinal cord regions and associated functions with a single electrode placement using numerous cohorts simultaneously.

Due to variability that exists among individuals with chronic SCI, neuroimaging [31] was necessary prior to mapping. Use of MRI-based modeling to visualize the paddle electrode’s physical location relative to spinal levels, including proximity to the conus, was key to effectively targeting the neural circuits related to bladder function. Different combinations of scES cohorts delivering specific functions enabled bladder management. The storage program included a cohort at the rostral end of the array for natural low-pressure filling, which targeted spinal circuitries for guarding reflexes that promote continence (sacro-lumbar intersegmental spinal reflex pathway: spinal projection between pelvic and hypogastric level inputs/outputs [33]). Effective parameters to achieve capacity within recommended guidelines for volume and pressure in adults are consistent with our mappings in rodents, with low-pressure fills and volume increases reflected by prolonged inter-contractile intervals with stimulation above the sacral region [18,20]. A second storage cohort was available to modulate systolic blood pressure to reduce potential occurrences of autonomic dysreflexia [42]. Previous data by our group demonstrated regulation of systolic blood pressure at maximal bladder capacity with CV-scES [16].

Emptying also required multiple cohorts, one of which was the cardiovascular cohort to minimize risk of autonomic dysreflexia from elevated detrusor pressure (necessary for voiding). Two other cohorts were used simultaneously for A70 and A243 targeted spinal circuits to (1) increase bladder pressure/contraction and (2) facilitate urine flow by modulating EUS activity while reducing urethral resistance. Based upon our prior mappings both in humans [16] and a rodent SCI model [19] for initiation of a void contraction, the sacral level containing the parasympathetic outflow via the pelvic nerve to the bladder [33] was targeted (bladder contraction cohort). At the same time, the purported spinal circuits that modulate the EUS (based upon rodent studies [20,43]) were targeted to overcome EUS overactivity, which is common post-SCI and enable increased urine flow. Performing both together enabled sufficient void efficiency with low residual volume. For A70, achieving low residual volumes (greater than 90% voiding efficiency) warranted continued use of bladder scES for daily management in replacement of daily catheterizations. Note that for our previous trial [16], only a single cohort targeting the pelvic circuitry for void was used. Although the prior study successfully achieved void initiation, as evidenced by elevated bladder pressure and contraction, void efficiency (assessed by post-void residual volume) remained significantly below the ≥90% efficiency threshold recommended by the International Continence Society. Consequently, participants were unable to transition from intermittent catheterization to scES-mediated voiding due to persistently high residual volumes. The present trial introduces a novel approach by simultaneously targeting a location at L3 that is part of a spinal network that is capable of modulating EUS activity (level known to contain the spinal ejaculation generator in humans [44]) in conjunction with sacral stimulation for void initiation (pelvic circuitry). This combined strategy is designed to improve voiding efficiency and reach a more normal physiological range (≥90% benchmark) for clinical translation.

Note that as found previously [16], utilization of sensations and voluntary effort (intent) was a critical component of void attempts (an active process during mappings; not passive), potentially utilizing intact residual descending projections from the pontine micturition center [33]. Relaxation of the EUS was evident with onset of intent in two of the three participants (see Table 3). Although participants are instructed not to strain, void attempts engage surrounding musculature, as the bladder does not exist in isolation. Deliberate micturition begins with a moderate increase in intra-abdominal pressure triggered by conscious contraction of the thoracic diaphragm, muscles of the abdominal wall, and levator ani muscle [45,46].

For A243, reported sensations of urgency and increased flow rates while catheterizing when BV-scES was on suggest successful mapping, despite the lack of a leak or void. The presence of bladder neck elevation and emptying while in a sitting position possibly explains the lack of successful emptying. The participant’s report of leakage only during his bowel program with digital stimulation and difficulties at times with catheter insertion are consistent with this explanation. The unanticipated observation of an elevated bladder neck underscores the value of small feasibility studies such as the present investigation, which can reveal findings that were not foreseen a priori. Future trials could minimize the impact of such occurrences by incorporating screening cystourethroscopy when clinically indicated and by including pre-existing structural abnormalities among the exclusion criteria. This study exclusion would not necessarily preclude SCI individuals with scES device implants from using a BV-scES program in the future, as the anatomical feature could be overcome in a different position, such as standing (simultaneous use of stand-scES cohort). Note that it is not known whether the elevated bladder neck developed after SCI, which would indicate an acquired rather than a congenital non-neurogenic condition.

Besides posture, other contributing factors impacting A243’s inability to void even with BV-scES include detrusor underactivity, detrusor–sphincter dyssynergia, the stimulation field, and lower limb activity. For example, Participant A243 experienced excessive muscle activity in the lower limbs during the void phase and Participant A124 presented excessive muscle activity during the fill phase, even at low volumes. We provided A124 with a muscle control cohort within the cardiovascular cohort, which relaxed the participants’ muscles, thereby enabling filling and the observed voids. This finding suggests that excessive muscle activity observed in A243 during the emptying phase may also have been a contributing factor to his inability to initiate void.

Participant A124, unlike Participant A70, did not benefit from the at-home activity-based recovery training, even though the initial void efficiency with BV-scES was higher after mapping. A noticeable difference was the lack of consistency in training. These observations suggest for the first time that repetitive training of the bladder may be needed for long-term efficacy of bladder scES, although other contributing factors (per A243 above) are possible.

Off-target positive effects reported in questionnaires included erectile, ejaculatory and bowel functions. For erectile activity, bladder scES inhibited function. These anti-erectile effects suggest a sympathetic-mediated mechanism (sacral parasympathetic pathways are pro-erectile) [47]. All participants reported an increase in reflexive erections, likely reflecting enhanced sensory input from the genital region [47] and consistent with observed improvements in sensation, although these were primarily reported in the context of bladder function.

In addition to bladder scES effects on erection, there was a re-emergence of lost ability to ejaculate, which occurred for A70 during training and has since continued. Noteworthy is that the L3 spinal level (targeted during fill and emptying phases) contains a well-documented spinal ejaculation generator in both animals and humans [44,48,49,50,51,52]. The reported benefit indicates the promising potential for direct targeting of the spinal ejaculation generator, which is positioned near or may even overlap with neurons of the purported EUS lumbar spinal coordination center, suggesting possible interaction and coordination [34]. In a pre-clinical study in a rodent model, L3-level scES was used to drive ejaculatory function in both intact animals and those having complete or incomplete injuries, with sensitivity further improved with intermittent locomotor training post-SCI [53]. Note that EMG responses depict the urethrogenital reflex in both the bulbospongiosus muscle and EUS [53].

Further participant-reported off-target effects of bladder scES involve improvements in bowel function. Improved timing and sensitivity for bowel management are consistent with findings from our prior animal and human studies demonstrating benefits associated with activity-based recovery training [54,55].

## 5. Conclusions

In conclusion, this pilot feasibility study demonstrates that lumbosacral epidural stimulation can support physiological bladder filling and voiding in individuals with spinal cord injury, with durable catheter-free voiding observed in one of three participants. By demonstrating consistent within-subject changes across time points, the results strengthen confidence in the observed effects and suggest meaningful functional improvements. Collectively, these outcomes contribute to advancing understanding in the field and may inform the design of larger, more rigorous studies as well as future clinical applications. Targeting both the bladder and EUS spinal networks permitted more efficient voiding, enabled by technological advancements of our team’s custom software controller, using multiple cohorts simultaneously and a smooth transition from storage to emptying phases within milliseconds. Study limitations include uncertain generalizability beyond a small pilot sample; the fact that all participants were white males and AIS A; the need for manual adjustment of intensity (e.g., adjusting CV cohort amplitude as the bladder approaches capacity); and the absence of sham control or blinding for scES ON/OFF conditions. The next step is development of a closed-loop, adaptive, real-time processing system that will automatically modulate stimulation programs based on acquired multimodal sensor data. The home and community daily use of an implanted multi-cohort spinal epidural stimulation device after chronic SCI to effectively store urine at safe pressures and efficiently empty after transferring to the toilet without the need for catheterization provides feasibility data for a larger-scale clinical trial and promise as an effective therapeutic intervention for bladder management.

Participant Perspective: Post-study testimonial from A70—“I came into this study hoping for any bit of feeling or movement I could get. The results were amazing. Prior to the stimulator I had no sensation when I had to go, or ability to void my bladder without the use of a catheter. Now I can feel when I have to go, and my bladder empties every time. It’s a great feeling to have feeling! After 13 years of injury, I’ll hopefully never have another urinary tract infection.”

## Figures and Tables

**Table 1:** Participant summary.

**Participant Demographics**										
Participant ID	Age (yrs)		Gender	Race	Injury Level		ASIA	Years Post Injury	Bladder Emptying Method *	
A70	40		Male	White	T3		A	12	CIC	
A243	38		Male	White	T5		A	9	CIC	
B253	36		Male	White	T4		A	21	CIC	
A124	31		Male	White	T7		A	9	CIC	
AIS ** Pre-Implant (Screening) Baseline										
	Sensory Score				Motor Score				Anal Sensation	Anal Contraction
Participant ID	LT, Left Side	PP, Left Side	LT, Right Side	PP, Right Side	Left Leg	Right Leg	Left Arm	Right Arm		
A70	27	20	20	20	0	0	25	25	No	No
A243	25	24	27	24	0	0	25	25	No	No
B253	24	22	23	22	1	1	25	25	No	No
A124	30	30	31	32	1	2	25	25	No	No

* CIC—Clean Intermittent Catheterization. ** Neurologic level of injury is derived from sensory and motor scores on the American Spinal Injury Association Impairment Scale. AIS grade A is defined as motor and sensory-complete injury. Sensory score includes sensation to light touch (LT) and pin prick (PP) and ranges from 0 to 48 for each side (left or right), with each of 12 segments (T10 to S5, with S4 and S5 counted as one dermatome) scored as 2 (normal sensation), 1 (impaired sensation), or 0 (absent). Motor scores include leg and arm muscle strength corresponding to L2 to S1 and C5 to T1 spinal segments. Each spinal segment is scored as 0 (total paralysis), 1 (palpable or visible muscle contraction), 2 (movement with gravity eliminated), 3 (movement against gravity), 4 (less than full power), or 5 (normal).

**Table 2:** Clinical renal characteristics of participants.

Renal Characteristics						
Participant ID	Left Kidney L/W/H (cm)	Left Kidney Parenchymal Thickness (mm)	Right Kidney L/W/H (cm)	Right Kidney Parenchymal Thickness (mm)	Bladder, L/W/H (cm)	Bladder Wall Thickness (mm)
A70 Pre-Implant	12.6/6.1/5.9	20.7	12.7/5.3/6.0	18.6	5.3/7.5/2.5	2.4
Post-Training	12.6/5.6/5.3	19.0	12.5/4.6/5.4	16.7	Normal	N/A
A243 Pre-Implant	10.9/-/-	19.9	11.3/-/-	15.1	N/A	1.4
Post-Training	10.9/-/-	11.5	11.0/-/-	12.0	N/A	2.4
A124 Pre-Implant	10.4/5.4/5.0	14.4	10.4/4.9/4.9	12.6	6.4/6.5/7.0	2.1
Post-Training	9.9/-/-	12.7	11.0/-/-	11.5	N/A	1.8

**Table 3:** Cystometry data summary.

ID	Condition/ Time Point	Capacity (mL)	Compliance (mL/cmH_2_O)	Max/End-Fill Pdet (cmH_2_O)	DO (Yes/No)	Void Volume (mL)	PVR (mL)	Void Efficiency (%)	DSD (Yes/No)
A70	Screening	666	92.5	10.1/7.2	No	0	666	0	No BCx
	Final Mapping, scES ON	587	33.4	27.7/17.6	Yes	87	500	15	Yes
	Post-Training, scES OFF	694	23.7	33.2/29.3	Yes	0	694	0	Yes
	Post-Training, scES ON	840	38.0	36.4/22.1	Yes	640	200	76	No
A243	Screening	553	45.3	16.2/12.2	Yes	0	553	0	No BCx
	Final Mapping, scES ON	786	50.4	23.1/15.6	Yes	0	786	0	No BCx
	Post-Training, scES OFF	632	140.4	16.7/4.5	No	0	632	0	No BCx
	Post-Training, scES ON	435	101.2	9.4/4.3	No	0	435	0	No *
A124	Screening	552	31.9	53.0/17.3	Yes	10	430	2	Yes
	Final Mapping, scES ON	401	7.6	71.4/52.7	Yes	251	145	63	No **
	Post-Training, scES OFF	504	11.4	50.2/44.4	Yes	96	408	19	Yes
	Post-Training, scES ON	484	16.1	52.0/30.1	No	36	447	7	Yes

* When program switched from BC-scES to BV-scES, a bladder contraction occurred which activated the guarding reflex. As soon as intent to void began, there was EUS relaxation (EMG recording). ** No bladder contraction, but there was EUS relaxation (void with intent was slow). BCx: bladder contraction; DO: detrusor overactivity; DSD: detrusor–sphincter dyssynergia; PVR: post-void residual.

**Table 4:** Lower Urinary Tract Data Set Questionnaire outcome summary.

ID	Time Point	Direct Signals	Indirect Signals *				
		Bladder Sensation	Fill-Evoked Spasms (Legs/Abdomen/Back)	Autonomic Dysreflexia Symptoms	# Cath’s (Day/Night)	# UTi’s **	Bladder Medication Usage
A70	Screening	No	No	No	4/1	2	No
	Post-Training	No	No	No	8/1	0	No
	Follow-up	Pressure	No	No	0-scES	0	No
A243	Screening	No	Yes	Yes	3–4/1–2	0	No
	Post-Training	Urgency	Yes	No (scES ON)	5–6/1–2	0	No
	Follow-up	Urgency	Yes	No (scES ON)	4–5/1–2	0	No
A124	Screening	No	Yes	Yes	3–4/0	2	No
	Post-Training	No	No (scES ON)	No (scES ON)	3/0	0	No
	Follow-up	N/A	N/A	N/A	N/A	N/A	N/A

* All 3 participants report penile shrinkage with bladder fullness. ** Total urinary tract infections (UTi’s) at screening time point estimated by participants for at least a year prior. # Cath’s reflects the number of clean intermittent catheterizations performed during waking hours and the number of sleep interruptions for catheterization.

**Table 5:** Home-training data summary.

ID	Sessions	Fill–Void Cycles with Measure of Void Volume		Fill–Void Cycles with Toilet Pee		Total Missed Sessions (i.e., No Recording)	Leaks (Count)
		Number of Cycles (Total)	Average Voided/Residual Volumes (mL)	Number of Cycles (total)	Average Residual Volume (mL)		
A70	1–5	15	252/170	0	N/A	0	1
	6–10	15	258/142	0	N/A	0	0
	11–15	19	309/100	0	N/A	0	0
	16–20	12	366/98	1	20	0	0
	21–25	5	446/80	6	96	0	0
	26–30	6	474/117	4	61	0	0
	31–35	3	453/23	11	44	0	0
	36–40	6	348/147	3	62	0	0
	41–45	0	N/A	5	20	2	0
	46–50	0	N/A	0	N/A	5	0
	51–55	0	N/A	1	60	4	0
	56–60	1	500/100	6	88	0	0
	61–65	1	325/60	7	67	0	0
	66–70	1	380/10	4	39	3	0
	71–75	2	400/30	1	30	4	0
A124	1–5	14	0/332	0	N/A	0	0
	6–10	13	76/350	0	N/A	0	0
	11–15	9	47/444	0	N/A	0	0
	16–20	10	40/440	0	N/A	0	0
	21–25	9	32/400	0	N/A	0	0
	26–30	10	44/410	0	N/A	0	0
	31–35	10	13/335	0	N/A	0	0
	36–40	10	34/465	0	N/A	0	0
	41–43	6	25/375	0	N/A	0	0

**Table 6:** Summary of off-target results.

**Bowel Function Data Overview**				
**ID**	**Time Point**	**Bowel Care**	**Neurogenic Bowel Dysfunction Score**	**Self-Report**
A70	Screening	Digital Stimulation; mini enema	9	None
	Post-Training	Digital Stimulation; mini enema	9	No changes
	Follow-up	Digital Stimulation; mini enema	9	Less digital stimulation
A243	Screening	Digital Stimulation; mini enema	14	None
	Post-Training	Digital Stimulation; mini enema	16	“More regular & consistent”
	Follow-up	Digital Stimulation; mini enema	16	“More feeling, get gas pain”
A124	Screening	Digital Stimulation; suppository	10	None
	Post Training	Digital Stimulation; suppository	10	“Quicker” Less Spasms
	Follow-up	N/A	N/A	N/A
**Sexual Function Data Overview**				
**ID**	**Time Point**	**IIEF Score Orgasmic Function (2–10)**	**Medication for Erectile Function**	**Self-Report**
A70	Screening	2	Viagra	None
	Post-Training	2	Viagra	Get more random erections
	Follow-up	7	Viagra	Can now ejaculate with vibration device
A243	Screening	6	TriMix	None
	Post-Training	6	TriMix	Erection & ejaculation “easier”
	Follow-up	7	TriMix	None
A124	Screening	2	None	None
	Post-Training	2	Viagra	Get more erections
	Follow-up	N/A	N/A	N/A

## Data Availability

Data are publicly available on the SPARC Portal (RRID:SCR_017041) at the following DOI: https://doi.org/10.26275/t2ve-vu9o.

## Supplementary Materials

The following supporting information can be downloaded at: https://www.mdpi.com/article/10.3390/jcm15166287/s1, Supplementary Figure S1: Intraoperative neurophysiological mapping during midline stimulation. (A) Schematic representing participant position in prone during neurophysiological mapping. The insert shows the approximate position of the epidural stimulation paddle over the lumbosacral spinal cord following laminectomy during stimulation delivery. The resulting electromyographic activity from multiple lower limb muscles (only three shown in the schematic) is recorded using established methodology [32]. (B) Shown in the schematic of the epidural stimulation paddle along with the cathode (red −) and anode (black +) contacts and resulting overlaid evoked potentials (black) and recruitment curves (blue). EMG recordings from left soleus (SOL) are noisy and removed from the final analysis. RF: Rectus Femoris; VL: vastus Lateralis; MH: Medial Hamstring; TA: Tibialis Anterior; MG: Medial Gastrocnemius; SOL: Soleus. Supplementary Figure S2: Post-operative neurophysiological mapping during midline stimulation. (A) Schematic representing participant position in supine during neurophysiological mapping. The insert shows the approximate position of the epidural stimulation paddle over the lumbosacral spinal cord. The resulting electromyographic activity from multiple lower limb muscles (only two shown in the schematic) is recorded using established methodology [32]. (B) Shown in the schematic of the epidural stimulation paddle along with the cathode (red −) and anode (black +) contacts and resulting overlaid evoked potentials (black) and recruitment curves (blue). Rectus Femoris; VL: vastus Lateralis; MH: Medial Hamstring; TA: Tibialis Anterior; MG: Medial Gastrocnemius; SOL: Soleus. Supplementary Figure S3: Relationship between epidural stimulation paddle placement and motor neuron distribution, and spatiotemporal mapping. (A) Schematic representing the precise location of the epidural stimulation paddle in relation to the vertebral levels, spinal segments and distribution of motor neuron pool supplying various lower extremity muscles along the lumbosacral spinal cord. For each motor neuron pool, darker and lighter shade represents major and minor distribution of the motor neurons, respectively [32]. (B,C) Shown in the schematic of the epidural stimulation paddle along with the cathode (red −) and anode (black +) contacts and heatmaps depicting peak-peak amplitudes corresponding to stimulation current (mA) and electrode configuration (numbers in each row refer to contacts of the electrode array tested shown to the left) from various lower limb muscles during midline and wide field stimulation. Rectus Femoris; VL: vastus Lateralis; MH: Medial Hamstring; TA: Tibialis Anterior; MG: Medial Gastrocnemius; SOL: Soleus.

## Abbreviations

The following abbreviations are used in this manuscript:

AIS	American Spinal Injury Association Impairment Scale
BC-scES	bladder capacity scES
BCx	bladder contraction
BV-scES	bladder void scES
CV-scES	cardiovascular scES
DO	detrusor overactivity
DSD	detrusor–sphincter dyssynergia
EMG	electromyography
EUS	external urethral sphincter
N/A	not applicable
PVR	post-void residual
scES	spinal cord epidural stimulation
SCI	spinal cord injury
UTi	urinary tract infection
