# Supplementary material for: A Pilot Feasibility Trial of Multi-Program Epidural Spinal Cord Stimulation for Bladder Function Recovery in Chronic Spinal Cord Injury
Source: J Clin Med. 2026 Aug 14;15(16):6287. doi: 10.3390/jcm15166287 (PMC13513323; doi:10.3390/jcm15166287)
Supplement: Supplementary file 1 [file jcm-15-06287-s001.zip › jcm-4387530-supplementary.pdf]

Supplementary Material

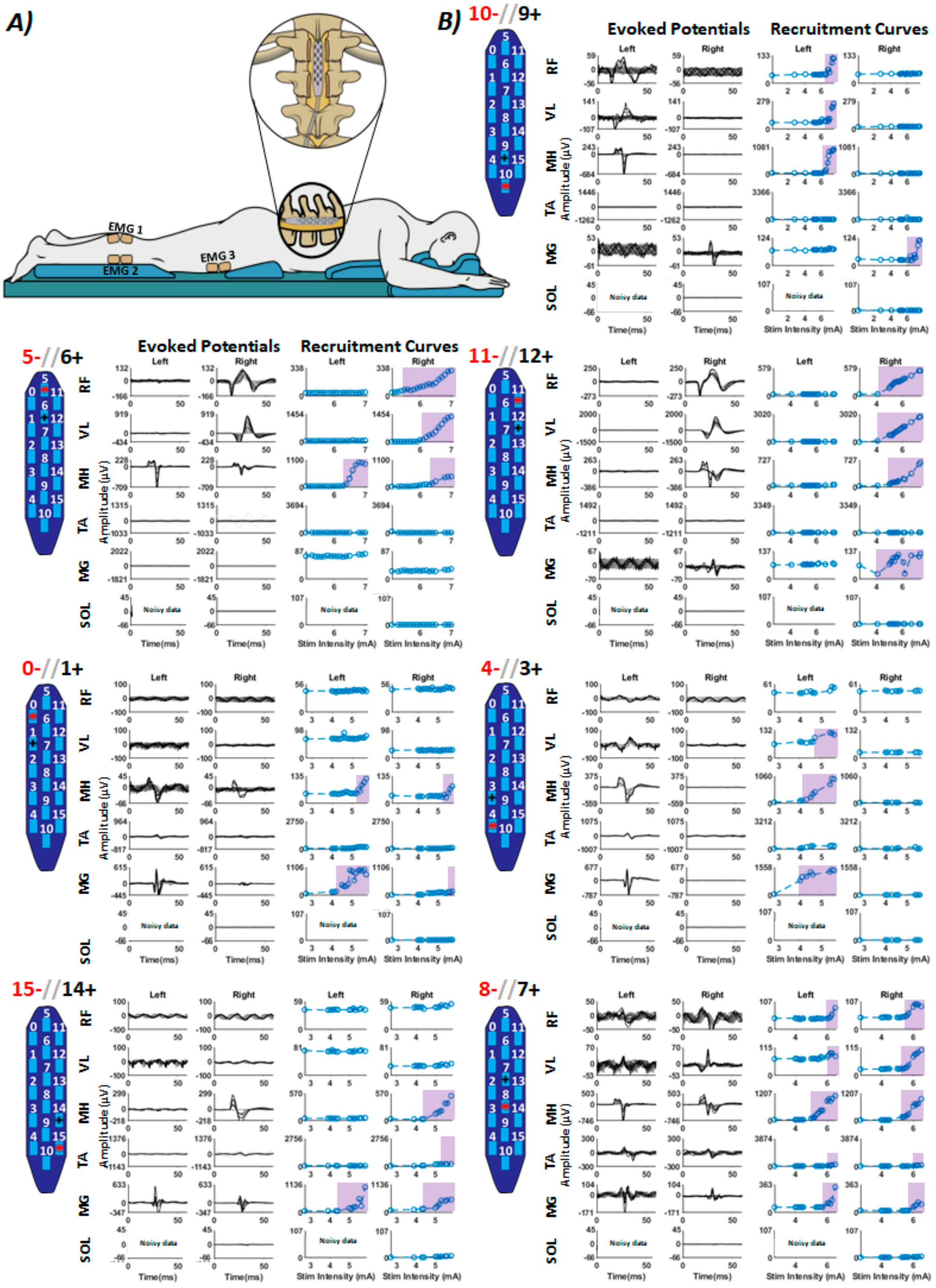

Supplementary Figure S1. Intraoperative neurophysiological mapping during midline stimulation. (**A**) Schematic representing participant position in prone during neurophysiological mapping. The insert shows the approximate position of the epidural stimulation paddle over the lumbosacral spinal cord following laminectomy during stimulation delivery. The resulting electromyographic activity from multiple lower limb muscles (only three shown in the schematic) is recorded using established methodology [32]. (**B**) Shown in the schematic of the epidural stimulation paddle along with the cathode (red -) and anode (black +) contacts and resulting overlaid evoked potentials (black) and recruitment curves (blue). EMG recordings from left soleus (SOL) are noisy and removed

from the final analysis. RF: Rectus Femoris; VL: vastus Lateralis; MH: Medial Hamstring; TA: Tibialis Anterior; MG: Medial Gastrocnemius; SOL: Soleus.

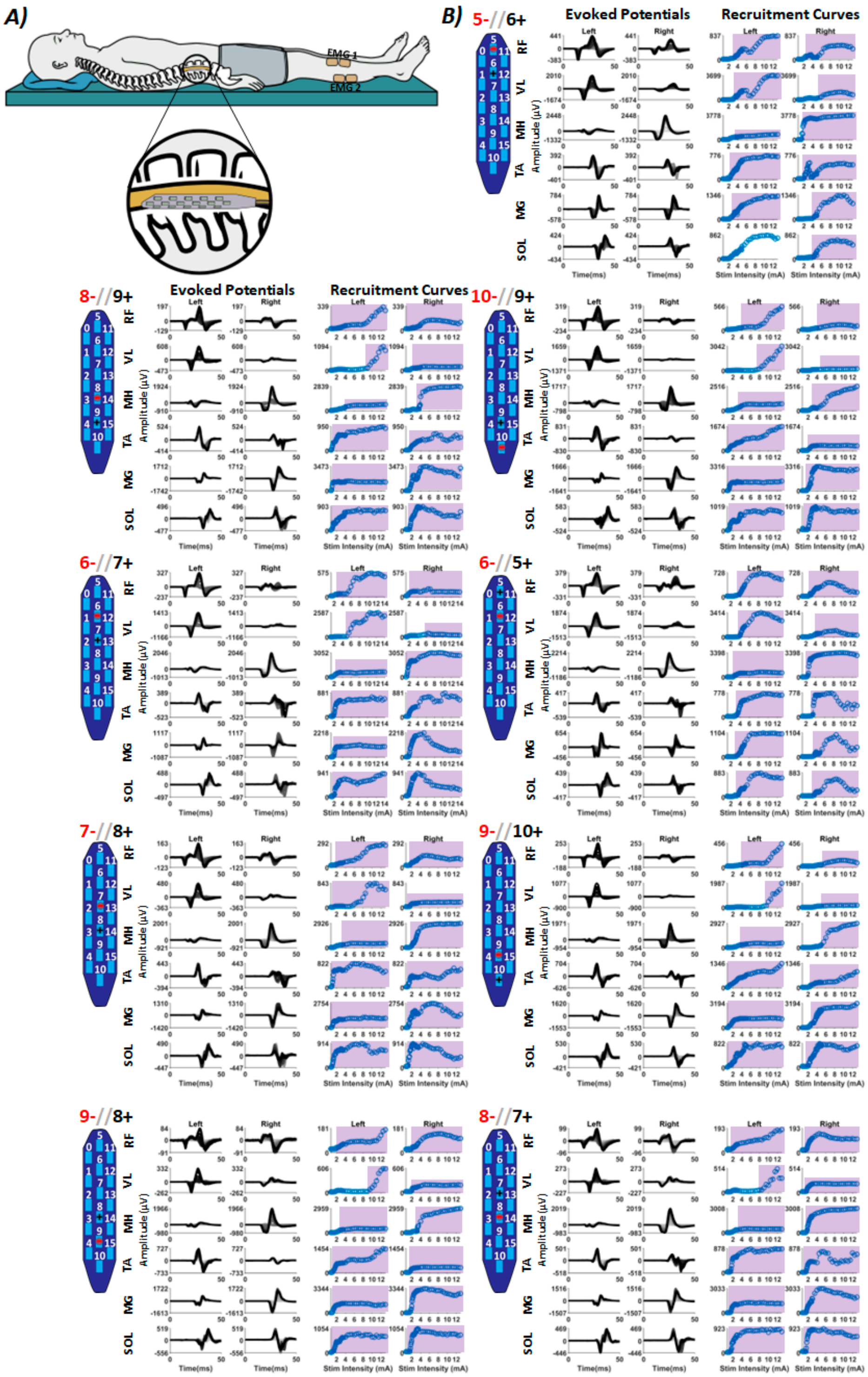

Supplementary Figure S2. Post-operative neurophysiological mapping during midline stimulation. (**A**) Schematic representing participant position in supine during neurophysiological mapping. The insert shows the approximate position of the epidural stimulation paddle over the lumbosacral spinal cord. The resulting electromyographic activity from multiple lower limb muscles (only two shown in the schematic) is recorded using established methodology [32]. (**B**) Shown in the schematic of the epidural stimulation paddle along with the cathode (red -)

and anode (black +) contacts and resulting overlaid evoked potentials (black) and recruitment curves (blue). Rectus Femoris; VL: vastus Lateralis; MH: Medial Hamstring; TA: Tibialis Anterior; MG: Medial Gastrocnemius; SOL: Soleus.

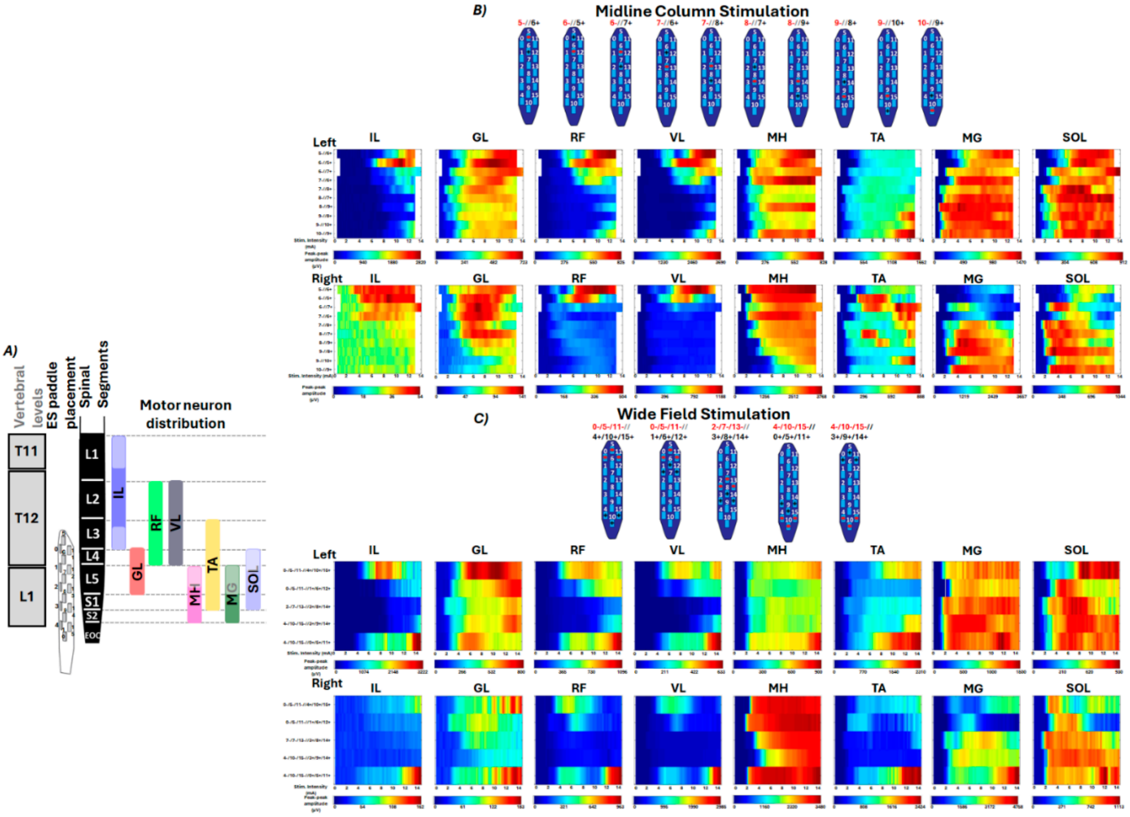

Supplementary Figure S3. Relationship between epidural stimulation paddle placement and motor neuron distribution, and spatiotemporal mapping. (**A**) Schematic representing the precise location of the epidural stimulation paddle in relation to the vertebral levels, spinal segments and distribution of motor neuron pool supplying various lower extremity muscles along the lumbosacral spinal cord. For each motor neuron pool, darker and lighter shade represents major and minor distribution of the motor neurons, respectively [32]. (**B,C**) Shown in the schematic of the epidural stimulation paddle along with the cathode (red -) and anode (black +) contacts and heatmaps depicting peak-peak amplitudes corresponding to stimulation current (mA) and electrode configuration (numbers in each row refer to contacts of the electrode array tested shown to the left) from various lower limb muscles during midline and wide field stimulation. Rectus Femoris; VL: vastus Lateralis; MH: Medial Hamstring; TA: Tibialis Anterior; MG: Medial Gastrocnemius; SOL: Soleus.
